# Evolutionary history and global spatiotemporal pattern of alfalfa mosaic virus

**DOI:** 10.3389/fmicb.2022.1051834

**Published:** 2022-12-21

**Authors:** Yanling Gao, Guoquan Fan, Shengqun Cheng, Wei Zhang, Yanju Bai

**Affiliations:** ^1^Industrial Crop Research Institute, Heilongjiang Academy of Agricultural Sciences, Harbin, China; ^2^College of Agronomy, Northeast Agricultural University, Harbin, China

**Keywords:** alfalfa mosaic virus, Bayesian phylodynamics, spatiotemporal transmission, geography-driven adaptation, positive selection, population dynamics

## Abstract

Alfalfa mosaic virus (AMV) is an important plant virus causing considerable economic loss to alfalfa production. Knowledge of the evolutionary and demographic history of the pathogen is limited but essential to the development of effective and sustainable pathogen management schemes. In this study, we performed worldwide phylodynamic analyses of AMV based on 154 nucleotide sequences of the coat protein gene, sampled from 1985 to 2020, to understand the epidemiology of this pathogen. Bayesian phylogenetic reconstruction estimates that the crown group of AMV dates back to 1840 (95% credibility interval, 1687–1955). We revealed that AMV continuously evolves at a rate of 4.14 × 10^−4^ substitutions/site/year (95% credibility interval, 1.04 × 10^−4^ − 6.68 × 10^−4^). Our phylogeographic analyses identified multiple migration links between Europe and other regions, implying that Europe played a key role in spreading the virus worldwide. Further analyses showed that the clustering pattern of AMV isolates is significantly correlated to geographic regions, indicating that geography-driven adaptation may be a factor that affects the evolution of AMV. Our findings may be potentially used in the development of effective control strategies for AMV.

## Introduction

Bayesian phylodynamic inference is recognized as one of the most extensively used methods in estimating how epidemics occur and in tracking their geographic spread, particularly RNA viruses, which rapidly accumulate genetic variation because of the lack of proofreading abilities by their replicases. Comprehensive analyses of the evolutionary dynamics of important pathogens can provide a view of the epidemiology and human-mediated spread of pathogens through time and space. To date, most phylodynamic studies have focused on key human RNA viruses, including influenza virus (Lam et al., 2008), dengue virus (Wei and Li, 2017), and SARS-CoV-2 (Lemey et al., 2020). However, the range of pathogens to which phylodynamic inference are applied is expanding. In recent decades, it has been applied to many important plant viruses such as tobacco mosaic virus (Gao et al., 2019), potato virus Y (Gao et al., 2020), and turnip mosaic potyvirus (Kawakubo et al., 2021). Understanding the evolution of emerging plant viruses can be of great importance for devising strategies to control the virus.

Alfalfa (*Medicago sativa* L.) or lucerne is a major forage crop that is cultivated worldwide. Alfalfa acreage in the world was maintained at a relatively stable level from the 1960s to 1980s. The cultivation area of alfalfa worldwide was 33 million ha in this period (Pan et al., 2017). However, this was followed by a sharp increase, reaching a peak in 1990.^1^ After the mid-2010s, the cultivation area of alfalfa decreased (Xie et al., 2021). However, North America remains the largest producer of alfalfa in the world, accounting for nearly 50% of the global production of alfalfa. Currently, the leading alfalfa producing countries are the United States, European Union, Argentina, Russia, Canada, and Australia. Comparatively, the leading alfalfa importing countries in descending order are the UAE, Saudi Arabia, Germany, Japan, and Jordan in 2020.^2^ However, the global production of alfalfa declined in recent years due to the SARS-CoV-2 pandemic.

Alfalfa mosaic virus, a species of the genus *Alfamovirus* within the family *Bromoviridae*, is an economically important pathogen in alfalfa worldwide (Hull, 1969; Lefkowitz et al., 2018). This virus has a wide host range, although its natural hosts mainly include the Fabaceae and Solanaceae families (Bol, 2003). AMV causes various mosaic, mottled, and calico blotching malformations, and necrosis (Hull, 1969; Jones et al., 2012), leading to crop losses of 14.8–22.8% losses in fresh weight and 15.0–18.1% in dry weight (Bailiss and Ollennu, 1986). It is transmitted by aphids in a non-persistent manner (Bol, 2003; Leur et al., 2019). Furthermore, AMV is transmitted mechanically and by plant seeds (Zhang and Guo, 1983; Jones and Pathipanawat, 1989; Valkonen et al., 1992; Jones and Coutts, 1996; He et al., 2010; Fidan et al., 2012). Moreover, weeds and cultivated plants also play an important role in AMV epidemiology because they serve as virus reservoirs and over-summer hosts for aphids, and thus enhanced the persistence and prevalence of AMV (Ormeño et al., 2006; Freeman and Aftab, 2011; Abdalla et al., 2020).

The genome of AMV consists of tripartite single-stranded positive-sense genomic RNA (RNA1, RNA2, and RNA3) and a subgenomic RNA 4 (Lefkowitz et al., 2018). RNA1 and RNA2 encode replicase subunits, P1 and P2, respectively. RNA 3 encodes the movement protein and the viral coat protein (CP) which is translated from the subgenomic RNA 4. The CP is thought to play an important role in the translational efficiency of RNAs, nucleolar and cytoplasmic shuttling, RNA-binding activity, virion formation, and systemic movement (Herranz et al., 2012). In addition, CP is commonly used as a molecular marker in the phylogenetic reconstruction of viruses belonging to the family *Bromoviridae* as well as other plant viruses (Codoñer et al., 2005; Duan et al., 2018; Guan et al., 2018; Xu et al., 2021).

Phylogenetically, all AMV isolates can be clustered into monophyletic groups. The separation seemed to correlate with differences in their geographic origins but not with differences in their host range or pathogenicity (Parrella et al., 2000). Based on restriction fragment length polymorphism analysis, Bergua et al. (2014) found that the AMV population was structured according to their geographic origin. Although there are many previous studies that focused on the genetic diversity and molecular evolution of AMV, most of the studies focused on the phylogenetic, recombination, and selection analyses at a national scale or on a specific host (Komorowska et al., 2021; Guo et al., 2022; Trucco et al., 2022). There are few studies on the evolutionary history and global spatio-temporal dynamics, yet may be valuable to the development of effective and sustainable pathogen management schemes.

In the present study, we conducted temporal dynamic, phylogeographic, and demographic history analysis on the 154 nucleotide sequences of the CP gene of AMV during the years from 1985 to 2020, to provide insights into the evolution and global spatiotemporal pattern of this pathogen.

## Materials and methods

### Alfalfa mosaic virus sequence dataset

**Figure 1:**
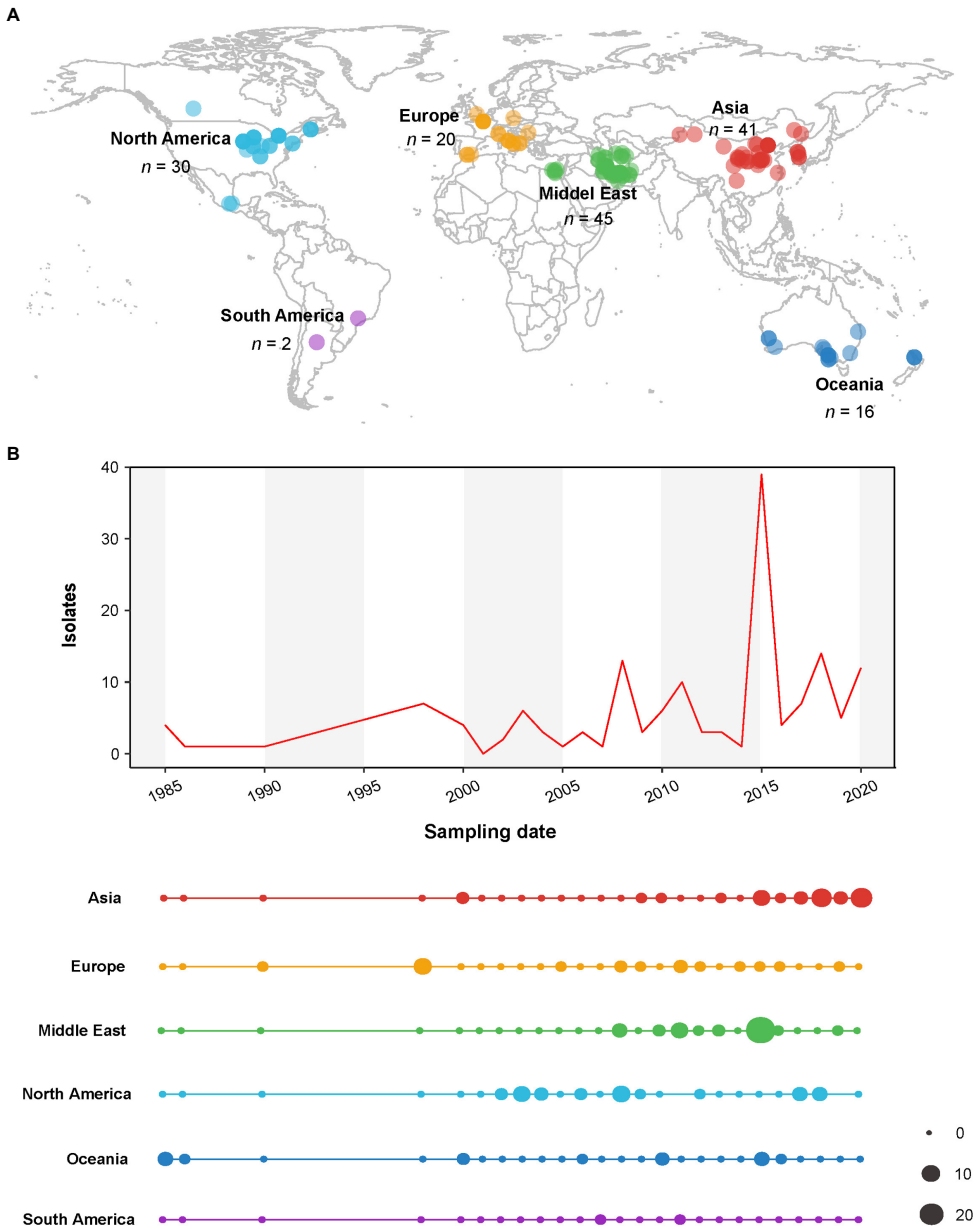
Spatial and temporal distribution of alfalfa mosaic virus (AMV) isolates used in this study. **(A)** Map illustrating geographic locations of AMV. **(B)** The total sample size and the distribution across the geographic locations over time (years). The dot sizes are proportional to the sample sizes.

One hundred and fifty-four nucleotide sequences of the CP gene of AMV isolates with known sampling dates and country of origins were downloaded from GenBank (Supplementary Table S1). The sampling regions and dates of these AMV isolates are presented in Figure 1. The viral isolates from 17 countries between 1985 and 2020 were geocoded and grouped into six populations according to their geographical origins: Asia (AS, *n* = 41), Europe (EU, *n* = 20), Middle East (ME, *n* = 45), North America (NAm, *n* = 30), Oceania (OC, *n* = 16), and South America (SAm, *n* = 2). We performed a codon-based alignment of the CP sequences with the MUSCLE algorithm (Edgar, 2004) implemented in MEGA X (Kumar et al., 2018).

### Recombination and likelihood-mapping analysis

To identify recombination signals within the CP sequences, we initially calculated the pairwise homoplasy index (PHI) using SplitsTree 4.13.1 (Huson, 1998) and subsequently screened for signals of recombination using the RDP4 suite (Martin et al., 2015), which provides formal testing for recombination using up to seven different algorithms: RDP, GENECONV, BOOTSCAN, MAXCHI, CHIMAERA, SISCAN, and 3SEQ. To minimize false identification, only the events detected by at least four algorithms were accepted, with an associated *p*-value of 10^−6^. No statistically significant evidence of recombination was observed in the data by either of the two methods; hence, the complete data set was used in subsequent analyses.

To examine the phylogenetic signal contained in the data set, a likelihood-mapping analysis (Strimmer and von Haeseler, 1997) based on the maximum likelihood was performed using the program IQ-TREE 2.13 (Nguyen et al., 2015) with 4,000 quartets randomly drawn. We found a fair amount of star-likeness, with 23.2% of all quartet points in region A (<33.0%, Supplementary Figure S1), indicating a stronger tree-like phylogenetic signal in our data set, which allowed for a reliable phylogeny inference.

### Assessing the temporal signal

To assess the clock-like behavior of our data set, we first calculated the correlation coefficients (*r*) of a regression of the root-to-tip genetic distance against the sampling time using TempEst (Rambaut et al., 2016). For this analysis, we estimated the tree topology and branch lengths using maximum likelihood analysis in IQ-TREE (Nguyen et al., 2015) with the SYM + I + G4 substitution model, which was selected based on the Bayesian information criterion calculated using ModelFinder (Kalyaanamoorthy et al., 2017) implemented in PhyloSuite 1.2.1 (Zhang et al., 2020). We then assessed the extent of the temporal structure using the recently developed BETS (Duchene et al., 2020). This approach compared the fit to data of two competing models, a heterochronous model (*M*_het_) and an isochronous model (*M*_iso_), using the marginal likelihood estimated by generalized stepping-stone sampling (Baele et al., 2016). For the *M*_het_, the data were accompanied by the heterochronous sampling time (with tip dates), and for the *M*_iso_, the actual sampling time was constrained to be contemporaneous (without tip dates). The best fit model to the data set was selected using Bayes factors (BF; Kass and Raftery, 1995). The results yielding a (log) BF log[P(Y|*M*_het_)]−log[P(Y|*M*_iso_)], and a value of at least five was considered as positive evidence for *M*_het_ over *M*_iso_, suggesting the presence of a sufficient temporal signal in the data.

### Bayesian phylogenetic analysis

To infer the evolutionary rate and timescale of AMV, we employed Bayesian coalescent approaches implemented in BEAST 1.10.4 (Suchard et al., 2018) under the best fit substitution model, as described above. To compare the fit of the constant size, exponential growth, and Bayesian skyline coalescent tree priors, we calculated marginal likelihoods using path and stepping-stone sampling (Baele et al., 2012). Therefore, this model combination was used for Markov chain Monte Carlo (MCMC) runs. The MCMC analysis was run for 200 million generations, with sampling every 20,000 generations. The sampling time of the sequences was used to calibrate the molecular clock during each run. Posterior distribution of the model parameters was estimated by sampling from the three independent Markov chains. Convergence of the chains was assessed using effective sample size values that were higher than 200 with Tracer 1.7 (Rambaut et al., 2018) and 10% of the sampling was discarded as burn-in.

### Discrete phylogeographic analysis

To infer the spatial–temporal spread of AMV across the world, we used a Bayesian stochastic search variable selection (BSSVS) model (Lemey et al., 2009) as implemented in BEAST to determine the asymmetric diffusion rates among localities designated as discrete geographical locations, which included Asia, Europe, Middle East, North America, and Oceania. The AMV population of South America was excluded from the BSSVS analysis due to an inadequate sample size (*n* = 2). The diffusion rates used to estimate rates of viral migration across the five geographic regions were calculated from the resulting log files using SpreaD3 (Bielejec et al., 2016). Significant migration pathways were determined based on the criteria of a BF > 3 (Kass and Raftery, 1995) and mean indicator >0.5 with the following categories: decisively supported diffusion, BF > 1,000; very strongly supported diffusion, 150 < BF < 1,000; strongly supported diffusion, 20 ≤ BF < 150; and supported diffusion, 3 ≤ BF < 20. The expected number of transitions at location state during ancestral history, relative to the data observed at tree distinct tips, was also calculated using Markov jump counts (Minin and Suchard, 2008).

To further analyze the inferred load and direction of migration through time, the maximum clade credibility tree from the MCMC analyses was read using Python scripts by Brynildsrud et al. (2018; https://github.com/admiralenola/globall4scripts). Here, migration events were presumed to occur at the nodes. This presumption may have resulted in a slight bias for inflated ages of AMV migration events.

### Demographic dynamics of alfalfa mosaic virus population

To reconstruct the demographic changes over time of AMV, a coalescent Bayesian skyline plot (BSP) was analyzed for the AMV populations of the five geographic regions, as well the combined population. For the separated populations, BSP analyses were implemented in BEAST 1.10.4 using an uncorrelated lognormal relaxed clock model with a uniform distribution for the prior of substitution rate of the CP gene, based on the previous estimate from the Bayesian phylogenetic analysis as described above, running 100 million steps sampled every 10,000 steps, with the first 10% discarded as burn-in.

### Phylogeny-trait association analysis

To identify the potential effects of geographical origin and host species on AMV evolution, we calculated three summary statistics (association index AI, parsimony score PS, and maximum monophyletic clade size MC) from the posterior tree samples using BaTS 2.0 (Parker et al., 2008). The AI and PS assess the association between traits (geographic origin and host species) and tree topology. The MC index assesses the association of traits to phylogeny. For BaTS analyses, we used phylogenetic uncertainty to evaluate phylogeny-trait correlations, using 1,000 random permutations of tip locations to calculate the null distribution for the AI, PS, and MC statistics.

### Episodic adaptive evolutionary selection

To examine specific sites for episodic adaptive evolutionary selection in the AMV, we employed an algorithm known as mixed effects model of evolution (MEME; Murrell et al., 2012) implemented in the Datamonkey server (Delport et al., 2010). In this analysis, two *ω* rate classes (a single *d*S value *α* and two separate *d*N values β^−^ and *β*^+^) were calculated using MEME per site with corresponding weights (the probability that the site evolved under each rate class at a specified branch). In the null model, *β*^−^ and *β*^+^ were constrained to be less than or equal to *α*, whereas in the alternative model *β*^+^ was not constrained. The fit of the models was compared using a likelihood ratio test (LRT). Episodic positive selection was inferred for the site when *β*^+^ > *α* at a site, and the alternative model provided a better fit than the null model.

## Results

### Temporal signal of alfalfa mosaic virus

For tip-dated analyses, data sets with a temporal structure are informative (Rieux and Balloux, 2016). Using TempEst, we found a weak correlation between tip dates and genetic distances (*r*^2^ = 7.07 × 10^−3^), suggesting the presence of various clock rates among lineages in our data set, and that a relaxed molecular clock might be most appropriate. Further BETS analysis results showed that the *M*_het_ yielded a higher log marginal likelihood (−5777.49) than the *M*_iso_ (−5782.85), implying that the *M*_het_ provided the best fit to our data set, and confirmed the data was considered to have an excellent temporal signal for subsequent dating analysis.

### Temporal dynamics of alfalfa mosaic virus

**Figure 2:**
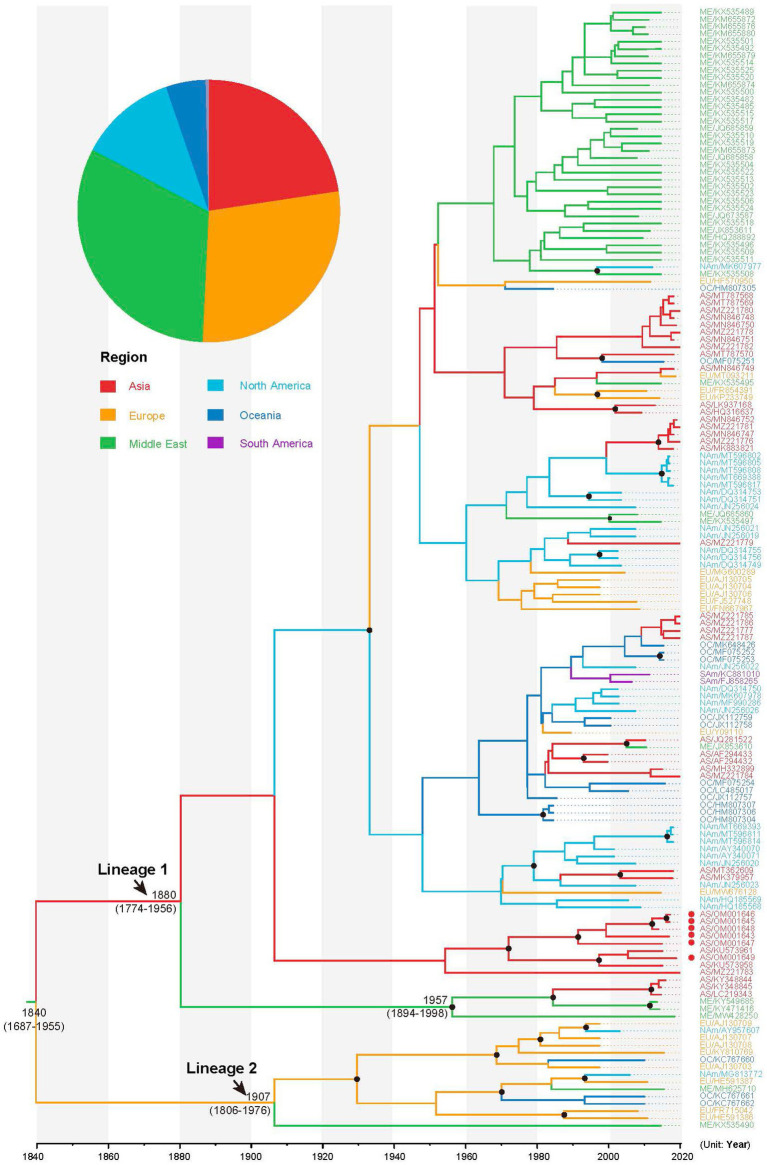
Maximum clade credibility tree inferred from the CP sequences of alfalfa mosaic virus (AMV). The tree topology has been chosen to maximize the product of node posterior probabilities and the tree branches have been color-coded according to their geographic origins. The inferred probability of the root for each geographic region is shown in the pie charts. Black circles indicate strong node support with posterior probability >0.95. AMV isolates sequenced in this study are indicated in red circles.

Using path and stepping-stone sampling, we confirmed a strong preference for a Bayesian skyline coalescent tree prior and uncorrelated lognormal relaxed clock model for the sequence data analysis (Table 1). Estimation using Bayesian phylogenetic analysis revealed that the mean substitution rate of the CP gene of AMV was 4.14 × 10^−4^ (95% credibility interval: 1.04 × 10^−4^ − 6.68 × 10^−4^) subs/site/year. The time scaled MCC tree revealed that AMV isolates comprised two lineages (Figure 2). Lineage 1 included viral isolates exhibiting considerable diversity among sampling regions, while lineage 2 contained 15 isolates, mainly from Europe. The results of our Bayesian phylogenetic analysis indicated that the crown group dates back to the 1840 CE (Common Era; 95% credibility interval, 1,687–1955) and the most recent common ancestors of AMV isolates in lineage 1 and lineage 2 were placed in 1880 (95% credibility interval, 1774–1956) and 1907 (95% credibility interval, 1806–1976), respectively. However, our Bayesian analysis did not provide support for a specific geographic location as a root node, with the Middle East (posterior probability (pp) = 0.32), Europe (pp = 0.28), and Asia (pp = 0.23) having similar posterior probabilities (Figure 2).

**Table 1:** Log marginal likelihoods of different combinations of the clock model and tree prior.

Molecular clock model	Coalescent tree prior	Path sampling	Stepping-stone sampling
Strict clock	Bayesian skyline	−6000.501	−6009.736
Strict clock	Constant size	−6039.014	−6051.125
Strict clock	Exponential growth	−6008.896	−6007.537
**Uncorrelated lognormal relaxed clock**	**Bayesian skyline**	**−5958.558**	**−5961.574**
Uncorrelated lognormal relaxed clock	Constant size	−5974.537	−5982.138
Uncorrelated lognormal relaxed clock	Exponential growth	−5974.656	−5981.063

The best-fitting tree prior and molecular clock model are indicated in bold font.

### Worldwide migration of alfalfa mosaic virus

**Figure 3:**
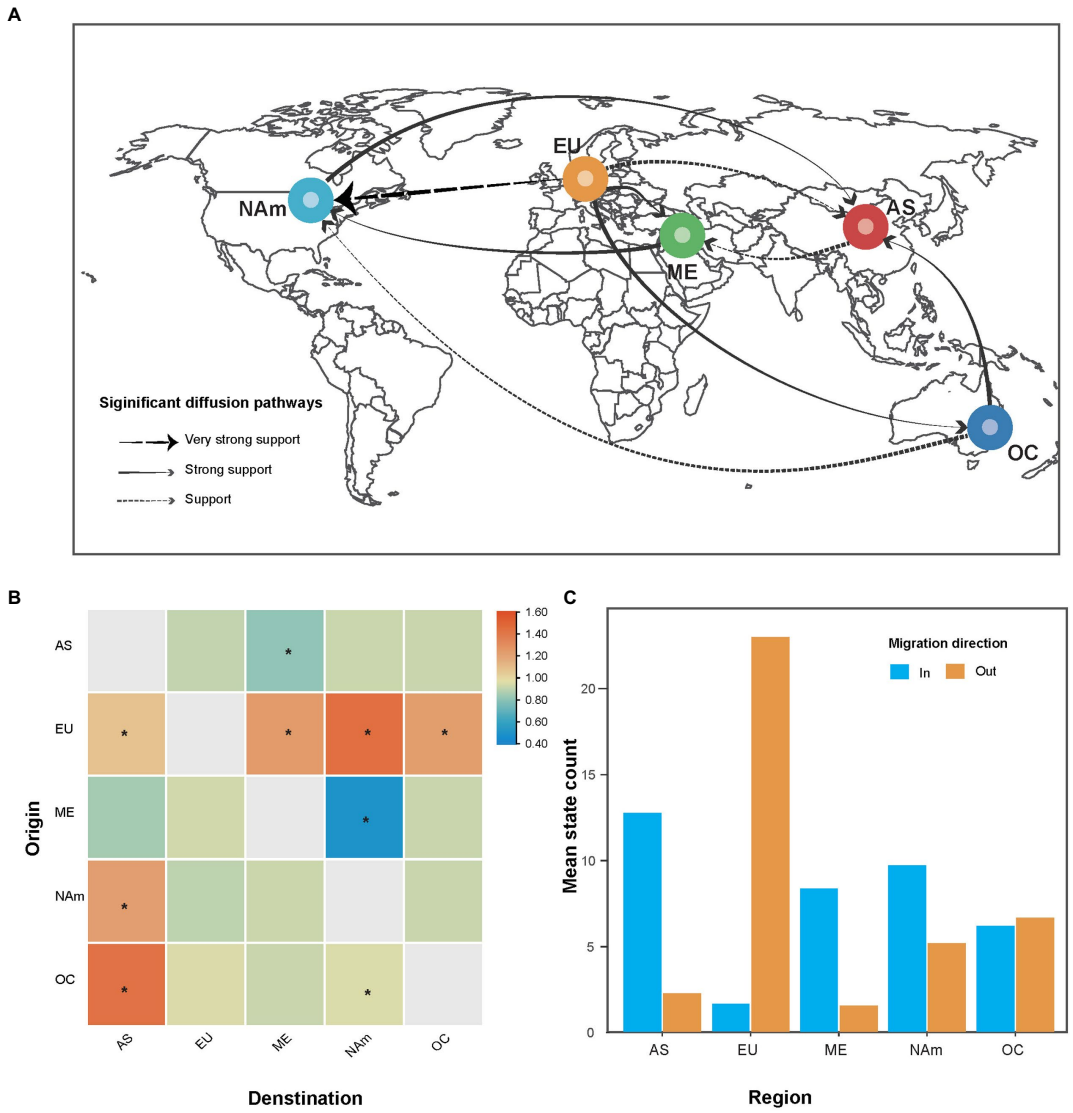
The spatial dynamics analysis of alfalfa mosaic virus (AMV). **(A)** Spatial diffusion pathway, **(B)** asymmetric migration rate matrix of AMV between each pair of regions, and **(C)** histograms of the total number of location-state transitions. Bayes factor (BF) > 3 and mean indicator of >0.5. Dashed black arrows, very strong support with 150 < BF < 1,000; solid grey arrows, strong support with 20 < BF < 150; and dashed grey arrows with BF < 20. The asterisks at the center of the cell indicate supported rates with 3 < BF < 20. AS, Asia; EU, Europe; ME, Middle East; NAm, North America; OC, Oceania.

Bayesian phylogeographic analysis suggested that nine migration links contributed to the diffusion of AMV throughout the world (Figure 3A). All the routes originating in Europe toward other regions showed significant support based on high BF values, implying that Europe plays a key role in AMV seeding across the world. Virus migration rates that we inferred were highest between Europe and North America with a mean rate of 1.44 (that is, migration events per lineage per year), followed by Oceania to Asia (a mean rate of 1.42). We observed the lowest mean migration rates from the Middle East to North America (Figure 3B), with a mean rate of 0.49. In addition, the total mean rate per Markov jumps for all regions supported the role of Europe as a seeding population. In contrast, it was shown that the in-migration of AMV was the greatest in Asia (Figure 3C).

### Spatial dynamics of alfalfa mosaic virus over time

**Figure 4:**
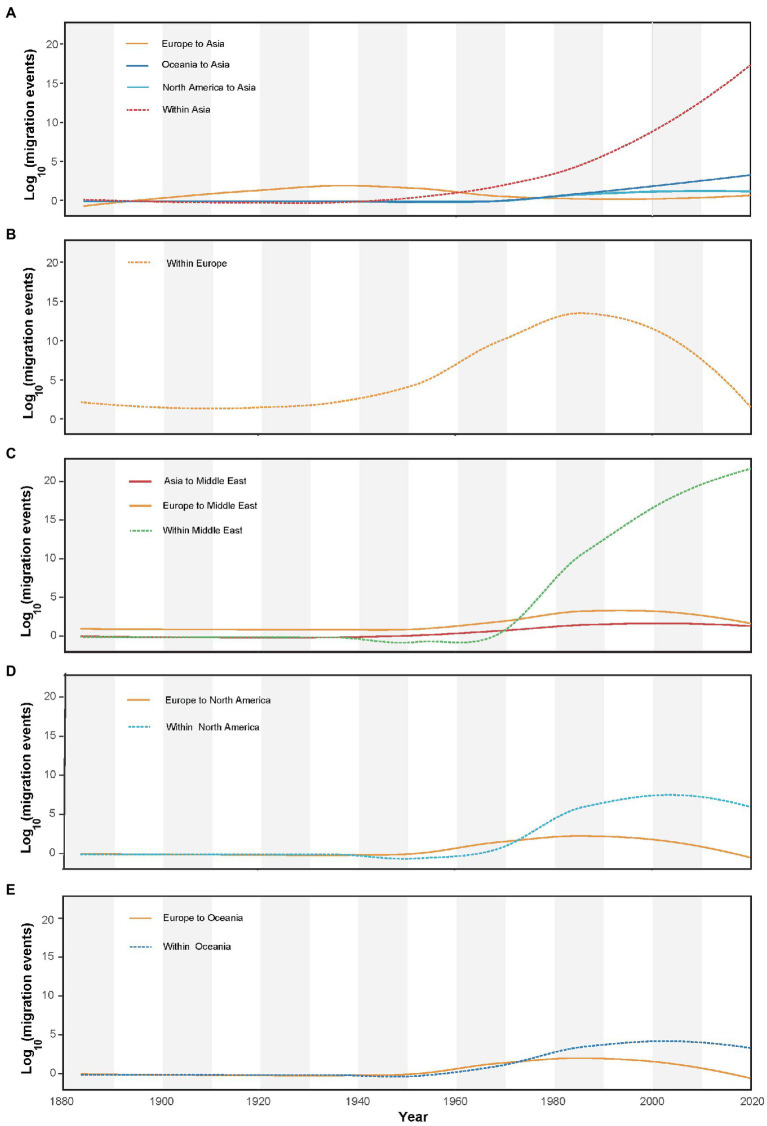
Inferred migration events (on log_10_ scale) of alfalfa mosaic virus through time (year). The plots also show within-region migration over time for **(A)** Asia, **(B)** Europe, **(C)** Middle East, **(D)** North America, and **(E)** Oceania.

AMV migration load and direction across time are summarized in Figure 4. Although a small wave of AMV migration from Europe to Asia had started before the early 1900s, the migrations from Europe to the Middle East, North America, and Oceania were not started until the early 1960s. Comparatively, a strong increase in prevalence of internal migration had been observed, particularly in Asia and the Middle East (Figures 4A,C) since the early 1940s. Despite the internal migration within Europe experiencing an increase before the 1980s, there was a sudden decline from the 1980s to the last sampling year (Figure 4B).

### Population dynamics of alfalfa mosaic virus

**Figure 5:**
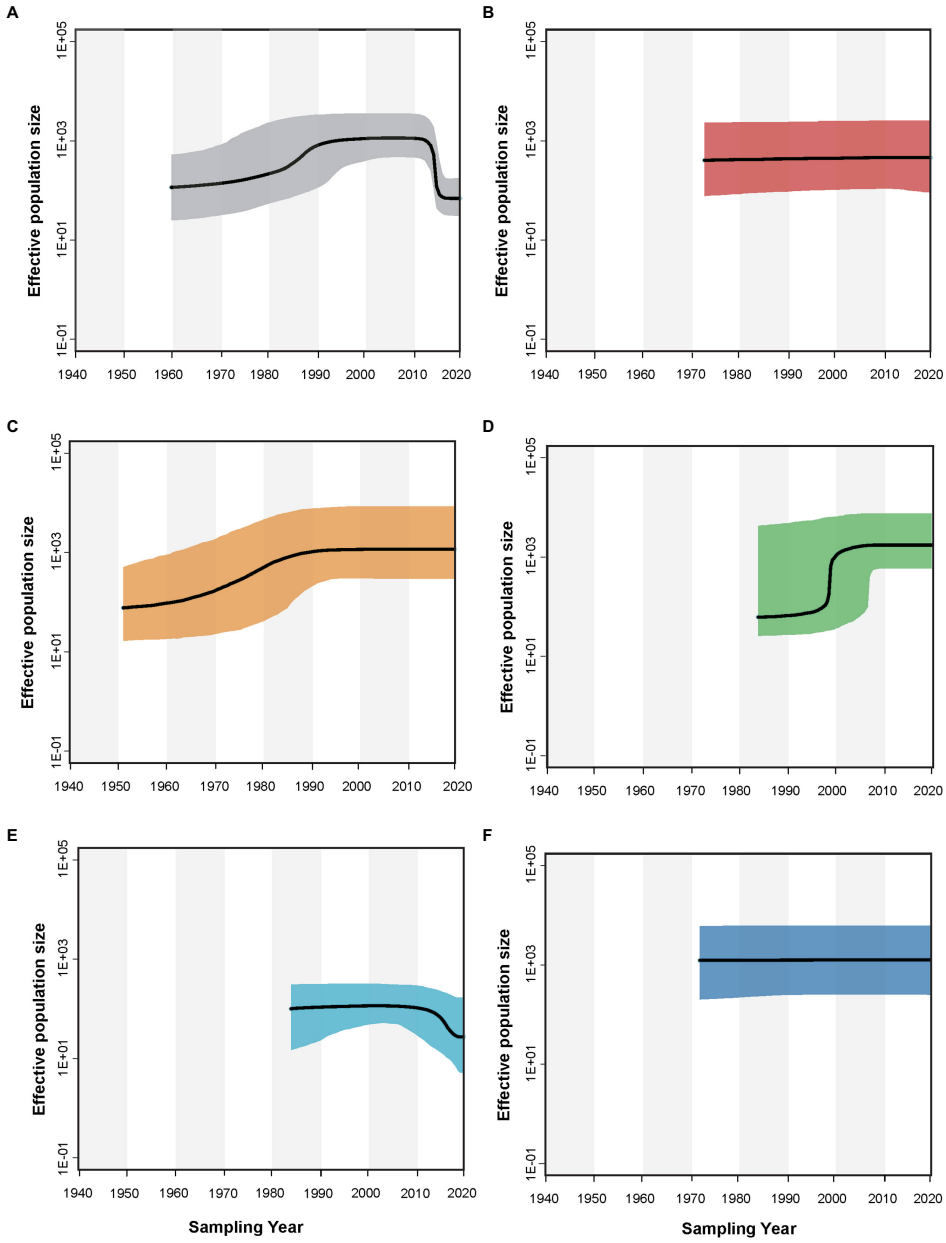
Bayesian skyline plot that describes the demographic history of alfalfa mosaic virus for **(A)** the global, **(B)** Asian, **(C)** European, **(D)** Middle Eastern, **(E)** North American, and **(F)** Oceanian AMV populations. The *y*-axis represents the population size (net) whereas the *x*-axis is given in years. The black lines show the median estimate of the population size, and the shaded areas show the 95% credibility interval.

Reconstruction of the demographic history by a Bayesian skyline plot indicated that the size of the AMV population changed with time (Figure 5). The global AMV population underwent a slight expansion between 1960 and 1990 and subsequently remained relatively constant, followed by a recent decline. AMV isolates originating from Europe and the Middle East underwent population expansion before a period of stability, while those from North America showed steady population size with a slight increase until the latest sampling year. In contrast, AMV from Asia and Oceania have maintained constant population sizes throughout the study time period.

### Adaptative evolution of alfalfa mosaic virus

When geographic regions were used as grouping factors, phylogeny-trait association analysis revealed that significant signals were found for the association between sampling regions and phylogenetic relationships (Table 2).

**Table 2:** Analysis of the geographical and host effects on the structure of AMV isolates.

Analysis	Isolate	Observed Mean (95% HPD)	Null Mean (95% HPD)	*p*-value
Region				
*AI*		3.03(2.42,3.65)	13.87(12.69,15.04)	<0.001^** *^
*PS*		33.03(30.00,36.00)	87.39(83.31,93.11)	<0.001^** *^
*MC* (Asia)	41	19.27(8.00,13.00)	2.36(1.96,3.00)	<0.01^**^
*MC* (Europe)	20	3.38(2.00,5.00)	1.57(1.07,2.11)	<0.01^**^
*MC* (Middle East)	45	22.71(13.00,34.00)	2.55(2.03,3.69)	<0.01^**^
*MC* (North America)	30	6.31(5.00,7.00)	2.07(1.41,3.00)	<0.01^**^
*MC* (Oceania)	16	53.70(3.00,6.00)	1.36(1.00,2.05)	<0.01^**^
*MC* (South America)	2	n/a	n/a	n/a
Host species				
*AI*		9.35 (8.36,10.31)	12.05 (11.05,13.04)	<0.001^***^
*PS*		62.73 (60.00,65.00)	70.12 (67.26,72.75)	<0.001^***^
*MC* (*Actinidiaceae*)	3	1.94 (1.00, 2.00)	1.03 (1.00, 1.07)	0.02^*^
*MC* (*Adoxaceae*)	1	n/a	n/a	n/a
*MC* (*Aphididae*)	3	1.17 (1.00, 2.00)	1.01 (1.00, 1.07)	1.00 ^ns^
*MC* (*Asclepiadaceae*)	1	n/a	n/a	n/a
*MC* (*Bignoniaceae*)	2	n/a	n/a	n/a
*MC* (*Caricaceae*)	1	n/a	n/a	n/a
*MC* (*Chenopodiaceae*)	3	1.00 (1.00, 1.00)	1.02 (1.00, 1.07)	1.00 ^ns^
*MC* (*Compositae*)	5	1.00 (1.00, 1.00)	1.02 (1.00, 1.08)	1.00 ^ns^
*MC* (*Cucurbitaceae*)	2			
*MC* (*Labiatae*)	5	1.00 (1.00, 1.00)	1.03 (1.00, 1.14)	1.00 ^ns^
*MC* (*Leguminosae*)	76	6.57 (5.00, 10.00)	4.13 (3.15, 5.98)	0.06 ^ns^
*MC* (*Malvaceae*)	2	n/a	n/a	n/a
*MC* (*Plantaginaceae*)	1	n/a	n/a	n/a
*MC* (*Polygonaceae*)	1	n/a	n/a	n/a
*MC* (*Portulacaceae*)	1	n/a	n/a	n/a
*MC* (*Solanaceae*)	40	3.99 (4.00, 4.00)	2.38 (1.99, 3.32)	<0.01^**^
*MC* (*Thripidae*)	4	1.18 (1.00, 2.00)	1.05 (1.00, 1.10)	1.00 ^ns^
*MC* (*Umbelliferae*)	3	1.92 (1.00, 2.00)	1.01 (1.00, 1.01)	<0.01^**^

AI, association index; PS, parsimony score; MC, maximum monophyletic clade; HPD, highest probability density interval; n/a, not available due to insufficient sample sizes (*n* < 3).

* Significance threshold: 0.01 < *p* < 0.05;

** Significance threshold: 0.001 < *p* < 0.01;

*** Significance threshold: *p* < 0.001.

However, with the exception of viral isolates from the host species of the *Actinidiaceae*, *Solanaceae*, and *Umbelliferae* families (*p*_MC_ < 0.05), no signal was observed between host species and phylogenetic relationships when the AMV isolates were clustered based on their host origins (Table 2). The BaTS results indicated an extensive geographical spatial variability of the pathogen, implying that geography-driven adaptation could be an important determinant of a factor during the evolution of AMV. Further, the results from the analysis of episodic adaptive evolutionary selection indicated that 13 out of 331 (3.92%, Table 3) of codon sites were found under positive selection (*p* ≤ 0.05).

**Table 3:** Sites under episodic selection detected by the MEME.

Site	*α*	*β* ^−^	*β* ^+^	LRT *p*-value	Substitution	
					From	To
9	0.00	0.00	2765.71	0.00	GGT (Gly)	TGG (Trp)
11	0.00	0.00	33.80	0.01	AAA (Lys)	GGA (Gly)
17	0.00	0.00	27.52	0.01	AAA (Lys)	ACA (Thr), CCA (Pro), AGA (Ronquist et al., 2012)
18	0.00	0.00	60.50	0.01	GGT (Gly)	CAT (His), AAC (Olsthoorn et al., 2004)
22	0.00	0.00	10.99	0.02	TAT (Tyr)	ATT (Ile)
23	0.00	0.00	38.38	0.00	GCT (Ale)	AAG (Lys), GGT (Gly)
100	0.00	0.00	12.27	0.04	ATA (Ile)	GTG (Val)
150	0.00	0.00	65.80	0.00	GCT (Ala)	ACT (Thr), CGT (Gly)
199	0.00	0.00	25.86	0.00	CGA (Ronquist et al., 2012)	GTA (Val)
203	0.00	0.00	7.95	0.02	CTC (Leu)	TCC (Ser)
205	0.00	0.00	20.90	0.00	AGT (Ser)	GAG (Glu), CGT (Ronquist et al., 2012)
208	0.00	0.00	63.87	0.00	ACT (Thr)	TTC (Phe), CCT (Leu), TCC (Ser)
213	0.50	0.00	181.46	0.01	GAC (Bol et al., 1971)	AAC (Olsthoorn et al., 2004), GCA (Ala)。

MEME, mixed effects model of evolution; α = synonymous substitution rate; β^−^ = non-synonymous substitution rate for the negative/neutral evolution component; β^+^ = non-synonymous substitution rate for the positive/neutral evolution component; LRT, likelihood ratio test.

## Discussion

We here investigated the molecular epidemiology of AMV based on the CP gene sequences of this virus and unveiled the comprehensive evolutionary history of the virus with a sampling window across 35 years.

AMV was first discovered in alfalfa (*Medicago sativa*) in America and was not officially named until 1931 (Weimer, 1931). Our phylogenetic analysis revealed that the most recent AMV common ancestor existed in 1840 (95% credibility interval 1687–1955; Figure 2), much earlier than that of the documented emergence of AMV. This can be explained by the fact that this disease has gone unnoticed for a long period.

Our phylogenetic analysis was unable to place the root of the tree in any particular geographic location because the Middle East, Europe, and Asia had similar posterior probabilities (Figure 2). One explanation is that the earlier AMV isolates were not sampled. In this study, the oldest AMV isolate was collected in 1985 (Supplementary Table S1). The other explanation is the multiple introductions of AMV due to pandemics. It is documented that there are two different origins of alfalfa, one the Mediterranean basin and the other Asia minor (Iran or Afghanistan; Hanson, 1972). Our phylogeographic analysis identified four migration pathways between Europe and other regions in the diffusion processes of AMV, indicating Europe might have played a key role in seeding the AMV epidemics. This finding is concordant with the global alfalfa trade during the past few decades, implying that AMV migration is associated with human-mediated activities. After North America, European countries have the second largest production of alfalfa in the world, with a cultivation area of nearly 2.5 million ha (Annicchiarico et al., 2015).

It is reported that geographic factors and host species play contributory roles in the evolution of many RNA viruses (Cuevas et al., 2012; Yu et al., 2021). Here, we hypothesized that deterministic events contributed to the current spatial population genetic structure, which is supported by the results of phylogeny-trait association analysis (Table 2). However, the results from the phylogeny-trait association analysis did not provide evidence for host-specific grouping of AMV isolates, except for the plant families *Umbelliferae*, *Actinidiaceae*, and *Solanaceae*, and we propose that to a certain degree, host-driven adaptation resulted in AMV diversification.

Drastic changes in population size coupled with demographic events may influence the generation, distribution, and maintenance of genetic variation. These effects not only act directly through genetic drift and mutation, but also act indirectly through impacts on migration and recombination as well as on the efficiency of natural selection to eliminate or amplify mutations (Wang and Whitlock, 2003). Our demographic analyses showed that the global population of AMV has remained small but has undergone recent expansion that may be associated with global alfalfa overproduction. Historical records indicate an oversupply of alfalfa on the market, reaching a peak of more than 33 million ha in 1990.^3^ In 2014, however, the cultivation area of alfalfa declined to 24 million ha (Xie et al., 2021). This is also concordant with our estimate of changes in AMV global population size (Figure 5), which suggests a correlation between the alfalfa cultivation area and the population size. It will be interesting to understand how human activities affect the demographic expansion of the AMV population.

It is notable that there are several limitations to the current study. For instance, AMV is a multipartite virus with a wide host range. There is evidence that the evolutionary stable equilibrium for the three genomic RNAs of AMV is host-species dependent (Wu et al., 2017). This suggests that a multi-gene data set is needed for a full appreciation of the evolution of AMV. Nevertheless, this study represents the first attempt to understand the global phylogeography of AMV, one of the most common viral pathogens of forage crops in the world.

## Conclusion

This study provides new insights into the evolutionary history of AMV based on Bayesian phylodynamic analysis of the CP gene data. We have identified multiple AMV migration pathways originating from Europe to other regions, suggesting that Europe is the major seeding region for the global spread of this pathogen. The dispersal patterns are likely to correlate with human activities. In addition, we found that geographically-driven adaptation may be an important determinant of the evolution of AMV. These results increase our knowledge about the evolution of AMV and may have potential implications for developing sustainable management strategies to control this pathogen.

## Data availability statement

The datasets presented in this study can be found in online repositories. The names of the repository/repositories and accession number(s) can be found at: https://www.ncbi.nlm.nih.gov/genbank/, OM001643, https://www.ncbi.nlm.nih.gov/genbank/, OM001644, https://www.ncbi.nlm.nih.gov/genbank/, OM001645, https://www.ncbi.nlm.nih.gov/genbank/, OM001646, https://www.ncbi.nlm.nih.gov/genbank/, OM001647, https://www.ncbi.nlm.nih.gov/genbank/, OM001648, https://www.ncbi.nlm.nih.gov/genbank/, OM001649, https://zenodo.org/record/7255913#.Y1np9OzP2ZY, 10.5281/zenodo.7255913.

## Author contributions

YB conceived the study. YG, GF, SC, and WZ performed the experiments. YG and YB analyzed the data and interpreted the results. YG and YB led the writing of the manuscript. All authors contributed to the article and approved the submitted version.

## Funding

This work was supported by grants from the China Agriculture Research System of MOF and MARA.

## Conflict of interest

The authors declare that the research was conducted in the absence of any commercial or financial relationships that could be construed as a potential conflict of interest.

## Publisher’s note

All claims expressed in this article are solely those of the authors and do not necessarily represent those of their affiliated organizations, or those of the publisher, the editors and the reviewers. Any product that may be evaluated in this article, or claim that may be made by its manufacturer, is not guaranteed or endorsed by the publisher.

## Footnotes

^1^ http://faostat.fao.org

^2^ http://faostat.fao.org

^3^ http://faostat.fao.org
