# Supplementary material for: Evolutionary history and global spatiotemporal pattern of alfalfa mosaic virus
Source: Front Microbiol. 2022 Dec 21;13:1051834. doi: 10.3389/fmicb.2022.1051834 (PMC9812523; doi:10.3389/fmicb.2022.1051834)
Supplement: Supplementary file 1 [file Table_1.DOCX]

**Table S1** Alfalfa mosaic virus isolates used in this study

| **No.** | **Isolates** | **Country** | **Pop. Code** | **Host** | **Collection year** | **Accession No.** |
| --- | --- | --- | --- | --- | --- | --- |
| 1 | HZ | China | AS | *Trifolium repens* L. | Oct-2009 | HQ316637 |
| 2 | AMV_China | China | AS | *Medicago sativa* L. | Nov-2010 | JQ281522 |
| 3 | XJA-1 | China | AS | *Medicago sativa* L. | Aug-2015 | KU573958 |
| 4 | XJA-2 | China | AS | *Medicago sativa* L. | Aug-2015 | KU573961 |
| 5 | ZN-1 | China | AS | *Nicotiana tabacum* L. | 09-Aug-2013 | LK937168 |
| 6 | AMV-Gyn | China | AS | *Gynostemma pentaphyllum* (Thunb.) Makino | Jul-2015 | MH332899 |
| 7 | AMV/hongyuan/MS | China | AS | *Medicago sativa* L. | Jul-2018 | MK379957 |
| 8 | AMV-Mint | China | AS | *Mentha haplocalyx* Briq. | Aug-2018 | MK883821 |
| 9 | BJMs1 | China | AS | *Medicago sativa* L. | 15-Sep-2018 | MN846747 |
| 10 | BJOl1 | China | AS | *Odontothrips loti* | 19-Sep-2018 | MN846748 |
| 11 | BJFi1 | China | AS | *Frankliniella intonsa* | 15-Sep-2018 | MN846749 |
| 12 | BJTt1 | China | AS | *Therioaphis trifolii* | 14-Jun-2019 | MN846750 |
| 13 | BJAp1 | China | AS | *Acyrthosiphon pisum* | 18-Sep-2018 | MN846751 |
| 14 | BJAc1 | China | AS | *Aphis craccivora* | 14-Jul-2019 | MN846752 |
| 15 | AMV-soybean | China | AS | *Glycine max* (L.) M. | 24-Jul-2018 | MT362609 |
| 16 | BJFi1 | China | AS | *Frankliniella intonsa* Trybom | 15-Sep-2018 | MT787568 |
| 17 | BJFi2 | China | AS | *Frankliniella intonsa* | 15-Sep-2018 | MT787569 |
| 18 | BJMs2 | China | AS | *Medicago sativa* L. | 15-Sep-2018 | MT787570 |
| 19 | AMV/China_Yangling/S | China | AS | *Medicago sativa* L. | 20-May-2020 | MZ221776 |
| 20 | AMV/China_Zhengzhou/H1 | China | AS | *Medicago sativa* L. | 13-Jun-2020 | MZ221777 |
| 21 | AMV/China_Yuanyang_1/H2 | China | AS | *Medicago sativa* L. | 23-Jun-2020 | MZ221778 |
| 22 | AMV/China_Yuanyang_2/H3 | China | AS | *Medicago sativa* L. | 30-Jun-2020 | MZ221779 |
| 23 | AMV/China_Yichuan/H7 | China | AS | *Medicago sativa* L. | 22-Jun-2020 | MZ221780 |
| 24 | AMV/China_Jiuquan/G | China | AS | *Medicago sativa* L. | 09-Jun-2020 | MZ221781 |
| 25 | AMV/China_Helinger/N1 | China | AS | *Medicago sativa* L. | 11-Jun-2020 | MZ221782 |
| 26 | AMV/China_Tumote_Left_Baaner/N2 | China | AS | *Medicago sativa* L. | 13-Jun-2020 | MZ221783 |
| 27 | AMV/China_Lankao/H4 | China | AS | *Medicago sativa* L. | 20-Jun-2020 | MZ221784 |
| 28 | AMV/China_Wenxian_1/H5 | China | AS | *Medicago sativa* L. | 21-Jun-2020 | MZ221785 |
| 29 | AMV/China_Wenxian_2/H6 | China | AS | *Medicago sativa* L. | 21-Jun-2020 | MZ221786 |
| 30 | AMV/China_Zhenping/H8 | China | AS | *Medicago sativa* L. | 24-Jun-2020 | MZ221787 |
| **31** | **GS 1** | **China** | **AS** | ***Solanum tuberosum* L.** | **24-Jun-2017** | **OM001643** |
| **32** | **GS 252** | **China** | **AS** | ***Solanum tuberosum* L.** | **8-Aug-2017** | **OM001645** |
| **33** | **GS 367** | **China** | **AS** | ***Solanum tuberosum* L.** | **21-Sep-2017** | **OM001646** |
| **34** | **GS 515** | **China** | **AS** | ***Solanum tuberosum* L.** | **11-Jul-2015** | **OM001647** |
| **35** | **HLJ 430** | **China** | **AS** | ***Solanum tuberosum* L.** | **25-Jun-2014** | **OM001648** |
| **36** | **NX 7** | **China** | **AS** | ***Solanum tuberosum* L.** | **7-Jul-2019** | **OM001649** |
| 37 | KR1 | South Korea | AS | *Solanum tuberosum* L. | 2000 | AF294432 |
| 38 | KR2 | South Korea | AS | *Solanum tuberosum* L. | 2000 | AF294433 |
| 39 | Gimcheon | South Korea | AS | *Trifolium repens* L. | 16-Jun-2016 | KY348844 |
| 40 | LS | South Korea | AS | *Ligularia stenocephala* Matsum. et Koidz | 24-Mar-2015 | KY348845 |
| 41 | Gomchi | South Korea | AS | *Ligularia fischeri*（Ledeb.）Turcz. | 24-Mar-2015 | LC219343 |
| 42 | AMV-PV1 | Czech | EU | *Trifolium pratense L.* | 2005 | MG600289 |
| 43 | Lye 80 | France | EU | *Lycopersicon esculentum* M. | 1998 | AJ130703 |
| 44 | Caa 1 | France | EU | *Capsicum annuum L.* | 1998 | AJ130707 |
| 45 | Dac 16 | France | EU | *Dacus carota* L. | 1998 | AJ130708 |
| 46 | Lyh 1 | France | EU | *Lycopersicon esculentum* M. | 1998 | AJ130709 |
| 47 | Restinclieres/2015 | France | EU | *Medicago sativa* L. | 01-Apr-2015 | MW676128 |
| 48 | 126 A | Italy | EU | *Portulaca oleracea* | 1998 | AJ130704 |
| 49 | 195 AN | Italy | EU | *Lycopersicon esculentum* M. | 1998 | AJ130705 |
| 50 | F 430 | Italy | EU | *Glycine max* (L.) M. | 1998 | AJ130706 |
| 51 | Lst | Italy | EU | *Lavandula stoechas* L. | 2009 | FN667967 |
| 52 | Tef-1 | Italy | EU | *Teucrium fruticans* | Mar-2011 | FR854391 |
| 53 | Ars2 | Italy | EU | *Araujia sericifera* | Apr-2012 | HF570950 |
| 54 | VIBtin | Italy | EU | *Viburnum tinus* L. | Nov-2014 | KP233749 |
| 55 | See-1 | Italy | EU | *Sechium edule* (Jacq.) Swartz | May-2019 | MT093211 |
| 56 | Danza | Italy | EU | *Lycopersicon esculentum* M. | 1990 | Y09110 |
| 57 | Aug-95 | Serbia | EU | *Medicago sativa* L. | May-2008 | FJ527748 |
| 58 | Tec1 | Spain | EU | *Tecomaria capensis*(Thunb.) Spach | Oct-2008 | FR715042 |
| 59 | TM2 | Spain | EU | *Hibiscus rosa-sinensis* L. | Jun-2011 | HE591386 |
| 60 | CV1 | Spain | EU | *Hibiscus rosa-sinensis* L. | Jun-2011 | HE591387 |
| 61 | FERA_160224 | UK | EU | *Nicotiana tabacum* L. | 2016 | KY810769 |
| 62 | / | Egypt | ME | *Solanum tuberosum* L. | Mar-2010 | HQ288892 |
| 63 | FRE | Egypt | ME | *Solanum tuberosum* L. | 2014 | KY549685 |
| 64 | CP2 | Egypt | ME | *Solanum tuberosum* L. | 15-Nov-2014 | KY471416 |
| 65 | Egyptian | Egypt | ME | *Ocimum basilicum* L. | 2016 | MH625710 |
| 66 | AMV-Eggplant-EG | Egypt | ME | *Solanum melongena* L. | 2019 | MW428250 |
| 67 | Ke.Ba.Po | Iran | ME | *Solanum tuberosum* L. | 6-Sep-2008 | JQ685860 |
| 68 | Ke.Si.Al | Iran | ME | *Medicago sativa* L. | 2-Aug-2008 | JQ685859 |
| 69 | Ke.Ba.Al | Iran | ME | *Medicago sativa* L. | 30-Nov-2008 | JQ673587 |
| 70 | Ke.Sh.Al | Iran | ME | *Medicago sativa* L. | 9-Jun-2008 | JQ685858 |
| 71 | PKH | Iran | ME | *Capsicum frutescens* L. | 12-Oct-2010 | KM655876 |
| 72 | SMZ | Iran | ME | *Solanum nigrum* L. | 21-Oct-2011 | KM655873 |
| 73 | WS | Iran | ME | *Chenopodium album* L. | 14-Jul-2011 | KM655879 |
| 74 | PSH | Iran | ME | *Solanum nigrum* L. | 22-Oct-2011 | KM655874 |
| 75 | PPB4 | Iran | ME | *Capsicum annuum* L. | 27-Jul-2011 | KM655880 |
| 76 | AK2 | Iran | ME | *Medicago sativa* L. | 27-Sep-2011 | KM655872 |
| 77 | AP1 | Iran | ME | *Medicago sativa* L. | 2011 | JX853610 |
| 78 | IRN-Tru | Iran | ME | *Campsis radicans* (L.) Seem. | 2012 | JX853611 |
| 79 | Fa.Es.A | Iran | ME | *Medicago sativa* L. | 31-Jan-2015 | KX535482 |
| 80 | Go.Mi.A | Iran | ME | *Medicago sativa* L. | 31-Jan-2015 | KX535485 |
| 81 | Ke.Sa.A | Iran | ME | *Medicago sativa* L. | 31-Jan-2015 | KX535489 |
| 82 | Kh.Be.A | Iran | ME | *Medicago sativa* L. | 31-Jan-2015 | KX535490 |
| 83 | Kh.Be.A2 | Iran | ME | *Medicago sativa* L. | 31-Jan-2015 | KX535492 |
| 84 | Kh.Bj.A | Iran | ME | *Medicago sativa* L. | 31-Jan-2015 | KX535495 |
| 85 | Kh.Ma.A | Iran | ME | *Medicago sativa* L. | 31-Jan-2015 | KX535496 |
| 86 | Si.Za.A1 | Iran | ME | *Medicago sativa* L. | 31-Jan-2015 | KX535497 |
| 87 | Si.Ze.A | Iran | ME | *Medicago sativa* L. | 31-Jan-2015 | KX535500 |
| 88 | Si.Zb.A | Iran | ME | *Medicago sativa* L. | 31-Jan-2015 | KX535501 |
| 89 | Te.Ka.A | Iran | ME | *Medicago sativa* L. | 31-Jan-2015 | KX535502 |
| 90 | Ya.Ha.A | Iran | ME | *Medicago sativa* L. | 31-Jan-2015 | KX535504 |
| 91 | Za.Es.A | Iran | ME | *Medicago sativa* L. | 31-Jan-2015 | KX535506 |
| 92 | Ha.Da.Po | Iran | ME | *Solanum tuberosum* L. | 31-Jan-2015 | KX535508 |
| 93 | Ke.Ke.Po | Iran | ME | *Solanum tuberosum* L. | 31-Jan-2015 | KX535509 |
| 94 | Ke.Ma.Po | Iran | ME | *Solanum tuberosum* L. | 31-Jan-2015 | KX535510 |
| 95 | Ke.Ma.Po2 | Iran | ME | *Solanum tuberosum* L. | 31-Jan-2015 | KX535511 |
| 96 | Ke.Ke.Pe | Iran | ME | *Capsicum annuum* L. | 31-Jan-2015 | KX535513 |
| 97 | Ke.Sa.Pe | Iran | ME | *Capsicum annuum* L. | 31-Jan-2015 | KX535514 |
| 98 | Es.Jo.Ch | Iran | ME | *Chenopodium album* L. | 31-Jan-2015 | KX535515 |
| 99 | Ke.Sh.Ch | Iran | ME | *Chenopodium album* L. | 31-Jan-2015 | KX535517 |
| 100 | Ch.Fa.Da | Iran | ME | *Dacus carota* L. | 31-Jan-2015 | KX535518 |
| 101 | Ho.Ba.So | Iran | ME | *Sonchus asper* (L.) Hill | 31-Jan-2015 | KX535519 |
| 102 | Kh.Sa.So | Iran | ME | *Sonchus asper* (L.) Hill | 31-Jan-2015 | KX535520 |
| 103 | Ke.Sh.Tr | Iran | ME | *Trogopogon sp.* | 31-Jan-2015 | KX535522 |
| 104 | Ke.Or.Am | Iran | ME | *Ammi majus* L. | 31-Jan-2015 | KX535523 |
| 105 | Ke.Si.Ru | Iran | ME | *Rumex acetosa* L. | 31-Jan-2015 | KX535524 |
| 106 | Kh.Sa.Pl | Iran | ME | *Plantago asiatica* L. | 31-Jan-2015 | KX535525 |
| 107 | NY-A 2002 | USA | NAm | *Medicago sativa* L. | 2002 | AY340070 |
| 108 | NY-B 2002 | USA | NAm | *Phaseolus vulgaris* L. | 2002 | AY340071 |
| 109 | Ca175 | Canada | NAm | *Solanum tuberosum* L. | 2003 | DQ314750 |
| 110 | Ca518 | Canada | NAm | *Solanum tuberosum* L. | 2003 | DQ314755 |
| 111 | Ca616 | Canada | NAm | *Solanum tuberosum* L. | 2003 | DQ314756 |
| 112 | 175 | Canada | NAm | *Solanum tuberosum* L. | Jun-2003 | MF990286 |
| 113 | Ca175-1 | Canada | NAm | *Solanum tuberosum* L. | Jun-2003 | MK607978 |
| 114 | Ca375 | Canada | NAm | *Solanum tuberosum* L. | 2004 | DQ314749 |
| 115 | Ca399 | Canada | NAm | *Solanum tuberosum* L. | 2004 | DQ314751 |
| 116 | Ca401 | Canada | NAm | *Solanum tuberosum* L. | 2004 | DQ314753 |
| 117 | CaM | Canada | NAm | *Solanum tuberosum* L. | Oct-2012 | MK607977 |
| 118 | Leo | Mexico | NAm | *Leonotis nepetaefolia* L. | Aug-2003 | AY957607 |
| 119 | Capsicum annuum cv. Ancho | Mexico | NAm | *Capsicum annuum* L. | Jul-2006 | MG813772 |
| 120 | Joe Davis | USA | NAm | *Glycine max* (L.) M. | 2006 | HQ185569 |
| 121 | Champaign | USA | NAm | *Glycine max* (L.) M. | 2008 | JN256019 |
| 122 | S01-18 | USA | NAm | *Glycine max* (L.) M. | 2008 | JN256020 |
| 123 | Rotation | USA | NAm | *Glycine max* (L.) M. | 2008 | JN256021 |
| 124 | Ar15 | USA | NAm | *Glycine max* (L.) M. | 2008 | JN256022 |
| 125 | SturdyII | USA | NAm | *Glycine max* (L.) M. | 2008 | JN256023 |
| 126 | C | USA | NAm | *Glycine max* (L.) M. | 2008 | JN256024 |
| 127 | SE12 | USA | NAm | *Glycine max* (L.) M. | 2008 | JN256026 |
| 128 | TN | USA | NAm | *Glycine max* (L.) M. | 30-Jun-2009 | HQ185568 |
| 129 | MO-2017 | USA | NAm | *Glycine max* (L.) M. | 24-Aug-2017 | MT596805 |
| 130 | OH-2017 | USA | NAm | *Glycine max* (L.) M. | 24-Aug-2017 | MT596802 |
| 131 | OH-2-2017 | USA | NAm | *Glycine max* (L.) M. | 24-Aug-2017 | MT669388 |
| 132 | IA-2017 | USA | NAm | *Glycine max* (L.) M. | 2-Oct-2017 | MT596808 |
| 133 | IA-1-2018 | USA | NAm | *Glycine max* (L.) M. | 18-Jul-2018 | MT596811 |
| 134 | IA-2-2018 | USA | NAm | *Glycine max* (L.) M. | 23-Aug-2018 | MT596814 |
| 135 | IA-3-2018 | USA | NAm | *Glycine max* (L.) M. | 23-Aug-2018 | MT596817 |
| 136 | IA-4-2018 | USA | NAm | *Glycine max* (L.) M. | 23-Aug-2018 | MT669393 |
| 137 | N20 | Australia | OC | *Medicago sativa* L. | 1985 | HM807304 |
| 138 | S30 | Australia | OC | *Medicago sativa* L. | 1985 | HM807305 |
| 139 | S40 | Australia | OC | *Medicago sativa* L. | 1985 | HM807306 |
| 140 | W1 | Australia | OC | *Medicago sativa* L. | 1985 | HM807307 |
| 141 | EW | Australia | OC | *Medicago sativa* L. | 1986 | JX112757 |
| 142 | Aq | Australia | OC | *Medicago sativa* L. | 2001 | JX112758 |
| 143 | Hu | Australia | OC | *Medicago sativa* L. | 2001 | JX112759 |
| 144 | 295 | Australia | OC | *Pisum sativum* L. | 2006 | LC485017 |
| 145 | SA-60 | Australia | OC | *Medicago sativa* L. | 10-Dec-2015 | MF075252 |
| 146 | AU-SA80 | Australia | OC | *Medicago sativa* L. | 10-Dec-2015 | MK648426 |
| 147 | SA-67 | Australia | OC | *Medicago sativa* L. | 10-Dec-2015 | MF075253 |
| 148 | QLD-215 | Australia | OC | *Medicago sativa* L. | 18-Dec-2015 | MF075251 |
| 149 | VIC-320 | Australia | OC | *Medicago sativa* L. | 19-Apr-2016 | MF075254 |
| 150 | 175 | New Zealand | OC | *Actinidia guilinensis* | 1-Aug-2010 | KC767660 |
| 151 | 176 | New Zealand | OC | *Actinidia glaucophylla* | 1-Aug-2010 | KC767661 |
| 152 | 178 | New Zealand | OC | *Actinidia fortunatii* | 1-Aug-2010 | KC767662 |
| 153 | Manfredi | Argentina | SAm | *Medicago sativa* L. | Dec-2011 | KC881010 |
| 154 | AMV_Brazil | Brazil | SAm | *Carica papaya* L. | 2007 | FJ858265 |

Isolates sequences in this study are shown in bold font.

AS, Asia; EU, Europe; ME, Middle East; NAm, North America; OC, Oceania; SAm, South America
