# Supplementary figures and images for: Evolutionary history and global spatiotemporal pattern of alfalfa mosaic virus
Source: Front Microbiol. 2022 Dec 21;13:1051834. doi: 10.3389/fmicb.2022.1051834 (PMC9812523; doi:10.3389/fmicb.2022.1051834)

Fig. S1

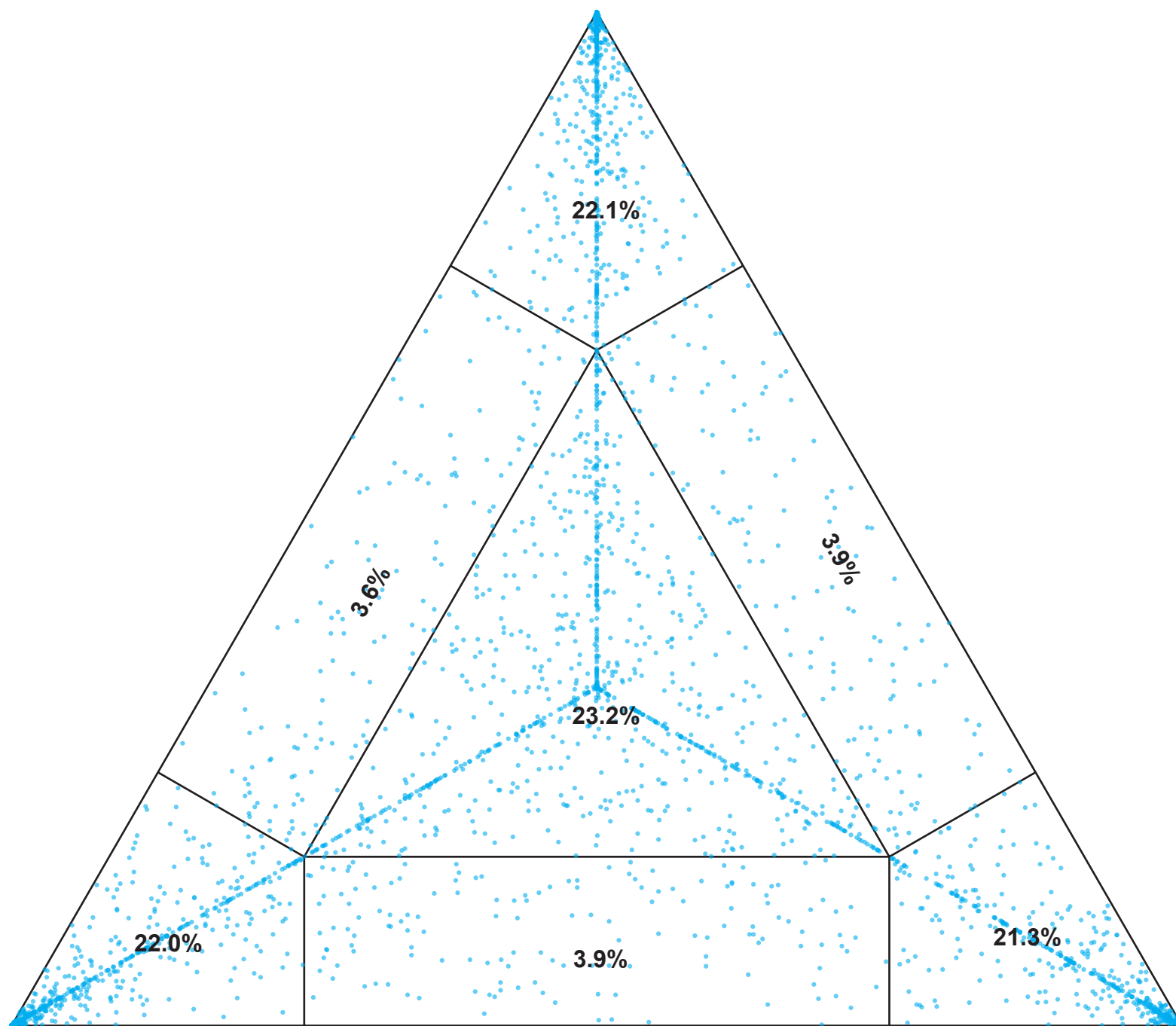

Supplement: Supplementary file 2 [file Data_Sheet_1.PDF]
